# Teleophthalmology and Artificial Intelligence As Game Changers in Ophthalmic Care After the COVID-19 Pandemic

**DOI:** 10.7759/cureus.16392

**Published:** 2021-07-14

**Authors:** Anna Nikolaidou, Konstantinos T Tsaousis

**Affiliations:** 1 Ophthalmology, Aristotle University of Thessaloniki, Thessaloniki, GRC; 2 Ophthalmology, Volos General Hospital, Volos, GRC

**Keywords:** teleophthalmology, artificial intelligence, covid-19, virtual clinics, machine learning

## Abstract

The current COVID-19 pandemic has boosted a sudden demand for telemedicine due to quarantine and travel restrictions. The exponential increase in the use of telemedicine is expected to affect ophthalmology drastically. The aim of this review is to discuss the utility, effectiveness and challenges of teleophthalmological new tools for eyecare delivery as well as its implementation and possible facilitation with artificial intelligence. We used the terms: “teleophthalmology,” “telemedicine and COVID-19,” “retinal diseases and telemedicine,” “virtual ophthalmology,” “cost effectiveness of teleophthalmology,” “pediatric teleophthalmology,” “Artificial intelligence and ophthalmology,” “Glaucoma and teleophthalmology” and “teleophthalmology limitations” in the database of PubMed and selected the articles being published in the course of 2015-2020. After the initial search, 321 articles returned as relevant. A meticulous screening followed and eventually 103 published manuscripts were included and used as our references. Emerging in the market, teleophthalmology is showing great potential for the future of ophthalmological care, benefiting both patients and ophthalmologists in times of pandemics. The spectrum of eye diseases that could benefit from teleophthalmology is wide, including mostly retinal diseases such as diabetic retinopathy, retinopathy of prematurity, age-related macular degeneration but also glaucoma and anterior segment conditions. Simultaneously, artificial intelligence provides ways of implementing teleophthalmology easier and with better outcomes, contributing as significant changing factors for ophthalmology practice after the COVID-19 pandemic.

## Introduction and background

**Figure 1:**
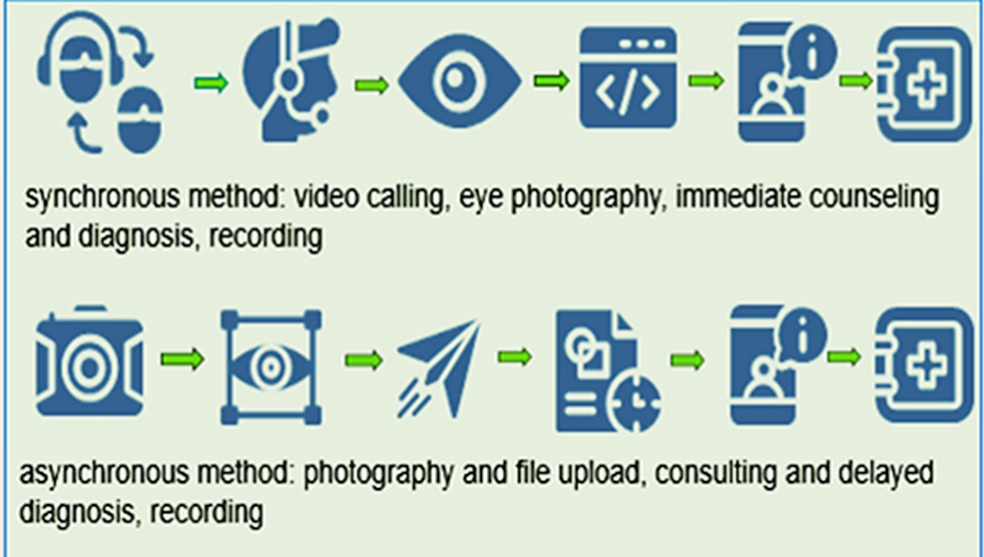
Synchronous and asynchronous methods of teleophthalmology.

As the SARS-COVID-19 pandemic arose in 2019, so did the implementation of telemedicine in a wide range of medical fields, among which, ophthalmology may be one of the most benefited ones [1]. “Telemedicine” means medicine from a distance, deriving from the Greek prefix “tele,” which means “from afar.” A subsection of telemedicine, teleophthalmology, involves telecommunication tools such as smartphones, powerful hardware, advanced software, wireless devices, and remote video tools but also applications of artificial intelligence (AI) [2-4]. There are two methods of exploiting teleophthalmology: synchronous and asynchronous, as shown in Figure 1. Between the two, the noticeable difference is the time required for diagnosis and consultation, while both result in the recording of data. Providing healthcare from a distance, teleophthalmology has in recent years been in the spotlight of modern medicine, which was further triggered by the pandemic [5].

In ophthalmology, a medical field that follows and benefits from technological advances, the evaluation and monitoring of patients remotely proves to be very helpful for both patients and health professionals. Teleophthalmology can benefit patients in a variety of ways by eliminating distances [6], reducing waiting times and intervals between visits and minimizing the risk of nosocomial infections for vulnerable groups. Thus, decongesting ophthalmology clinics also gives the opportunity to healthcare providers for better patient screening and more complete data recording [7]. Among others, diabetic retinopathy (DR), age-related macular degeneration, glaucoma, cataract and premature retinopathy are diagnosed early and monitored through devices and applications of AI [8,9].

Teleophthalmological practices can also be of great importance for circumstances like natural disasters, infectious disease outbreaks and, in general, whenever, and wherever healthcare is limited or inaccessible like in rural areas, remote villages and poorly resourced regions [10-14]. It has also been reported that even soldiers can benefit from teleophthalmology, avoiding unnecessary evacuation from warzone according to Cocozza et al. [15].

However, concerns arise about the immediacy of the procedure and the quality of the doctor-patient relationship that is likely to deteriorate. The application of teleophthalmology requires training in the use of devices by health professionals, which can be a burden for its wide use. Furthermore, patient accessibility is questioned especially for elderly patients who are less familiar with the technology. These challenges can trigger the effective use of teleophthalmology, which profits the established lifelong ophthalmological examination, but it cannot replace it even if its sensitivity and specificity prove to be of high value [16,17].

Undoubtedly, the SARS-COVID-19 pandemic constitutes a period that ophthalmological consultation that can be challenging due to the short distance between the ophthalmologist and the patient. Therefore, patients with chronic eye diseases that need monitoring and routine screening and patients that require follow-up visits are examples of cases that can avoid the hospital environment that comes with high viral exposure and use telemedicine [18]. Additionally, teleconsultation can be useful for the health professionals that are quarantined due to potential exposure to the virus, offering the chance to keep a close eye on their patients from a distance [19].

## Review

Diseases - implementation

1. *Retinal Disorders*

Image-based investigations are a crucial part of both retinal disorders and teleophthalmology implementations. Thus, the field of the retina is probably the one that teleophthalmology has benefited the most for both screening and follow up. For adult patients, DR and for pediatric patients, retinopathy of prematurity (ROP) are at the heart of teleophthalmological advances and applications.

The growing need for evaluation of retinal diseases is a key sector that teleophthalmology can thrive in, using synchronous and asynchronous methods for consultation. De Fauw et al. showcased that AI has the potential to be very useful for retinal diseases. An enormous number of images can be generated and processed with deep learning techniques [20]. Advanced fundus imaging techniques and optical coherence tomography (OCT) imaging have enabled the growth of retinal teleophthalmology systems, where remote imaging containing important clinical information is centrally interpreted [21]. Careful and proper integration of the retinal telemedicine programs in the current clinical practice will be significant for their proper use and the creation of virtual clinics in the medical retina may change the eye care delivery soon, as displayed in El-Khayat et al. [22,23].

Recent studies have shown that teleophthalmology can be an accurate and sensitive tool for screening in DR and is promising to be a universal newborn screening tool [24,25]. Additionally, Kozak et al. [26] highlighted in a prospective, interventional cohort study that remotely a retina specialist can also implement interventions such as telephotocoagulation for diabetic macular edema.

(a) Diabetic retinopathy (DR): It constitutes one of the most important complications of diabetes mellitus and is the leading cause of visual disability for DM patients. It is estimated that 486 million people worldwide have DM and almost ⅓ suffers from DR while the visual-threatening cases are increasing according to Yau et al. [27,28]. In this setting, teleophthalmological tools are essential for screening, management and follow up of DR. Retinal imaging is achieved with portable screening devices such as mobile imaging units, smartphone-based retinal photography and applications of AI providing high sensitivity and specificity [4,29,30]. Images are either evaluated by a remote ophthalmologist or automatically analyzed by a deep learning algorithm [31]. Also, diabetic macular thickening can be predicted through color fundus photographs from deep learning models demonstrated by Arcadu et al. enhancing the efficiency of DM diagnosis [32].

Screening with teleophthalmological means can have a significant impact on reducing vision loss caused by DR. Nationwide-based systems but also community-based programs are proving to boost the screening rates and result in avoiding visual complications around the world [33]. Tozel et al. completed a review of large DR telemedicine screening programs for DR across the globe and demonstrated that a high level of clinical accuracy and easier patient access are accomplished [34]. Unnecessary referral minimizing and cost-saving are also reported in recent studies for both rural population and urban community teleophthalmology services [35,36].

In several city-specific studies, such as Vujosevic et al. in Padova and Invernizzi et al. in Milan, (single-centre) teleophthalmology screening for DR proved to be convenient and well-received by patients and resulted in avoiding unnecessary referrals and less costs than in the standard slit-lamp examination [37, 38]. In the United Kingdom, the nationwide teleophthalmological screening program has been described as a great opportunity for DR patients that are least likely to attend a screening test [39]. Likewise, in Canada, both urban and rural programs of teleophthalmology have benefited geographically disparate populations and urged the guidance of healthcare delivery across the country [40]. Furthermore, in Zimbabwe Retinopathy Telemedicine Project (ZRTP), teleophthalmological screening gave an opportunity to limited-access patients to not only manage DR but also educate themselves on DR [41].

Widespread implementation of teleophthalmology for DR has been in the spotlight also in the USA, as the growing number of people having diabetes mellitus requires many specialists and screening visits. The teleophthalmological approach can annihilate distances and provide a wide range of specialists for consultation or automated consultation. However, as with every new technology, there are barriers to overcome, economic and utility and familiarity as Liu et al. explained, especially in rural settings [11,42]. In Chile, the use of telemedicine resulted in a boost/surge for screening coverage of DR patients and helped to overcome the lack of specialists as seen in Avendano-Veloso et al. [43].

As for imaging methods, Silva et al. compared DR identification between non-mydriatic ultrawide field (UWF) imaging and non-mydriatic multi-field fundus photography (NMFP) in a large ocular telehealth program and demonstrated significant benefits of the UWF imaging method [44]. The “reading” of the retinal images can be done by many health professionals like optometrists, general ophthalmologists and retinal specialists without significant variability, but with the training needed to improve consensus on image grading as Liu et al. highlighted [45]. As for the imaging, that can also be done by nonphysicians [46]. Pharmacy-based teleophthalmology was suggested by Coronado et al. in a study conducted in semi-urban areas of Ontario, Canada, choosing pharmacies as a strategic place that DM patients visit to acquire medications and proved to be effective on wide screening but not cost-effective compared to slit-lamp examination [47].

Follow-up care after the screening of DR is required to have effective screening and as demonstrated by Boucher et al. in a cross-sectional retrospective descriptive study, a high number of compliances was achieved in an urban community-based DR teleophthalmology project with a 91.9% compliance rate. Also, a high patient satisfaction score was achieved, which is also required for effective screening [48].

(b) Age-related macular degeneration (AMD): It is the main cause of visual impairment and blindness in Europe and the USA [49,50]. Almost 67 million people across Europe suffer from AMD and while the life expectancy is increasing, and by 2050 this number is expected to reach 77 million [49]. The lack of a well-defined and specific high-risk population for AMD is a limitation for the successful implementation of teleophthalmology for AMD. Teleophthalmological means AMD may require the extension of the remote screening tool kit to a network-connected fundus camera that includes technologies such as OCT and possibly OCT angiography.

Brady et al. reviewed the state-of-the-art telemedicine programs for AMD: some added screening for AMD in the already-existing DR telemedicine programs with positive results while others found AMD telemedicine programs to be ineffective economically [51]. Kawaguchi et al. in 2018 demonstrated that teleophthalmological programs for screening AMD are equally effective as the traditional slit-lamp examinations while having a statistically significant higher possibility of patient participation [52]. The “Muranga teleophthalmology study” in Kenya showcased a fair agreement of slit-lamp examination and web-based teleophthalmology assessment in both rural and urban settings. Additionally, 99% of patients were satisfied with the great majority preferring teleophthalmology than slit-lamp examination [53]. A randomized clinical trial in Canada proposed the benefit of teleophthalmology for health professionals working in such programs to expand their knowledge in retinal services under the teleophthalmic guidance of retinal specialists and highlighted the need for such programs in remote areas so that timely diagnosis of AMD and its monitoring are accessible for everyone [54].

The model of “virtual” follow-up appointments proved to be greatly helpful to contend with capacity issues as resulted in a single-center study by Tsaousis et al.: decreasing the time between appointments, improving the number of injections/patients/years, and most drastically boosting mean visual acuity (VA) improvement >15 letters [55]. Self-monitoring with a mobile application for AMD, proposed by Schmid et al., showed reasonable accuracy to detect wet AMD and classify dry vs. wet AMD. This kind of application also provides valuable data for patients monitoring through the years that are crucial for diagnosis and management [56].

(c) Retinopathy of prematurity (ROP): It constitutes a leading cause of preventable childhood blindness or visual impairment worldwide [57]. This proliferative disorder affects premature infants, usually of low birth weight. Inadequate screening is considered to be the cause of the high prevalence of ROP instead of the continuous advances in treatment like anti-vascular endothelial growth factor (anti-VEGF) agents and photocoagulation [58]. Teleophthalmology with fundus photography and remote grading by experienced ophthalmologists can drastically change the percentage of access to ophthalmic care for patients, especially in poorly resourced communities. When compared to binocular indirect ophthalmoscopy examination, telemedicine grading of dilated fundus imaging, no statistically significant difference in the sensitivity was detected in a multicenter study conducted by Biten et al. Both methods showed high inter-examiner variability [59]. Smartphone-based fundus photography systems are suggested by researchers across the world as the “need of the hour” for ROP proving to be of high feasibility and accuracy [60], cost-effective and user-friendly [61]. With a rising incidence of ROP and a limited number of adequately trained ophthalmologists, the need for an easy-using, fast and effective tool for screening is urgent [62,63]. Offering high image quality and affordability of imaging systems, telemedicine constitutes a valuable solution for vulnerable pediatric patients [64].

AI could not be missing from teleophthalmology instruments for ROP. Brown et al. proposed an algorithm using deep convolutional neural networks (CNN) to diagnose plus disease in ROP with comparable or better accuracy by human experts. Infants at risk of ROP in resource-limited or remote areas can benefit from such algorithms, using technology to prevent, detect and manage ROP [65]. In a recent review, Scruggs et al. highlighted the advantage of AI in the ROP diagnosis offering objectivity and improved accuracy (as a paradigm-shifting strategy to improve efficiency) [66]. Hu et al. proposed deep CNN are to identify the presence and severity of ROP disease per-examination using an automated analysis strategy. The model was proved to be effective with the only limitation being the falsely recognized reflection of light, as the ridge of ROP, resulting in an incorrect prediction outcome [67].

2. *Glaucoma*

Glaucoma constitutes one of the leading causes of irreversible blindness worldwide affecting mostly women and Asians [68]. Screening, diagnostic consultation and long-term treatment monitoring of glaucoma overcome the barriers of distance when using telemedicine [69]. Aiming to high-quality, cost-effective care to achieve the appropriate balance between clinical, economic and humanistic objectives, telemedicine is needed due to insufficient drug or intervention methods as presented in Delgrado et al. [70]. In LVPEI Glaucoma Epidemiology and Molecular Genetic Study, a rural population-based study, teleophthalmoscopic grading of peripheral anterior chamber depth with Van Herick (vH) technique showed that combined vH grading and ocular biometry increased the predictability of a gonioscopically occludable angle [71].

A pilot program in the USA using e-health to monitor low-risk glaucoma suspects achieved high patient follow-up compliance, proving telemedicine to be promising for glaucoma suspects [72]. Another study in the USA evaluated the accordance between teleglaucoma consultation and in-person assessment for glaucoma progression. The results showed high similarity between the two methods, suggesting teleophthalmology as an auxiliary tool to decongest clinics but with limitations due to a lack of absolute agreement [73].

Diagnostic accuracy, screening effectiveness and evaluation of progression risk can be facilitated by AI technologies using deep learning algorithms as presented by Mursch-Edlmayr et al. Specificity and sensitivity for OCT imaging, fundus photography and visual field (VF) testing are increased when using AI. For glaucoma, AI can predict progression earlier than conventional methods, but incorporation into the health system is needed so that it makes the most out of the benefits of AI [74]. AI can act as a form of teleophthalmological care for glaucoma, collecting data from devices that the patients use at home such as self-monitoring of intraocular pressure tonometry [75], smartphone-based head-mounted perimeter for detection of VF defects [76], ophthalmoscopic applications for smartphones and processing this data to result in a diagnosis or progression risk percentage [77]. Effective triage of the patients and facilitation of glaucoma clinical practice constitute fields that telemedicine is expected to thrive when Glaucoma AI algorithms meet regulatory approval [78].

3. *Pediatric Eye Disease*

The use of telemedicine can help to reduce delays in referrals and developing new specialized centers with teleophthalmology services is thought to be the future for pediatric ophthalmology in which timely referral can avoid vision permanent loss [79]. VA was evaluated with a phone application called GoCheckKids and the results showed a modest correlation of VA compared to a regular clinic protocol showing potential for future VA evaluation at home [80]. Video-recorded screening for assessing children was proposed by Sabri et al. in a study for pediatric amblyopia and eye disease that trained non-eye-care professionals can record adequate videos for a remote ophthalmologist to consider when there is no direct access for consultation [81].

As for postoperative care, teleophthalmology can also contribute to virtual visits for pediatric patients, which can be more pleasant for the children because of decreased waiting times with quality care maintained [82].

AI for pediatric eye disease is yet to be developed as for the adults, but significant advances have been reported as “classification of pediatric cataracts, prediction of postoperative complications following cataract surgery, detection of strabismus and refractive error, prediction of future high myopia, and diagnosis of reading disability.” Applications of AI are expected to pave the way for optimal clinical care for children with early disease detection and grading but most essentially widely accessed care [83].

4. *Anterior Segment Conditions*

Keratoconus, infectious keratitis, refractive surgery, corneal transplant, adult and pediatric cataracts, angle-closure glaucoma, iris tumors, post-refractive surgery keratitis and post-cataract surgery endophthalmitis constitute anterior segment conditions that could benefit from teleophthalmology and its AI applications but the efficiency of this is yet to be widely investigated [84-86].

Smartphone-assisted anterior segment imaging was performed in Iran with 10 and 90 Diopter lenses commercially available in ophthalmic settings, proving to be a useful alternative when a slit lamp is not available or the patient is bedridden or hospitalized [87]. In rural India in the EyeSmart study-I, teleophthalmology has been presented as a promising tool for the management and diagnosis of anterior segment diseases but also for the lid and adnexal pathologies. A pyramidal model was used for eye care delivery, in which vision technicians photographed patients' eyes and then teleconsultation was conducted if needed. This model with the use of EyeSmart application proved to overcome the barriers of distance, time and costs, for mostly lens-related (38.3%) followed by ocular surface pathologies (30.2%), lid and adnexa-related pathologies (8.6%), and corneal pathologies [86].

The application of AI to the diagnosis and management of cataracts was recently extensively analyzed by Ting et al. [88], but its applications in teleophthalmology are still not extensively researched. Integration of AI technology with telemedicine healthcare systems for anterior segment diseases requires large sample studies to ensure accuracy. Wu et al. presented a deep learning algorithm, ResNet, for the assessment and diagnosis of adult and pediatric cataracts of varying severities in a real-world setting. This algorithm, when combined with teleophthalmological tools (ocular surface images taken by cell phones, applications for clinical history and VA) and referral to community-based healthcare facilities and the tertiary eye hospitals, showed optimal diagnostic performance in grading (AUC > 0.99) [89].

5. *Emergencies*

Ocular fundus photography with teleophthalmological means can bring patients to medical attention at an earlier stage of disease, such as intracranial hypertension (IH). If easy access to ophthalmological care is not provided, many patients may not present to an ophthalmologist until IH has irreversibly altered their vision. As presented in a case report by Medert et al., the initial detection of idiopathic intracranial hypertension-associated papilledema in a male was made with the use of a comprehensive teleophthalmology screening examination, probably much earlier than it would have been if he had sought medical care in person [90]. An Italian study that scrutinized if smartphone-based ocular fundus photography could be used for the Emergency Department (ED) for hypertensive emergencies and compared it with traditional retinal fundoscopy, showed the decreased duration of examination and superior reliability of the smartphone-based method. This way, many missed diagnoses of acute hypertension emergencies can be avoided, and they can be conducted faster [91].

Coverage gaps in emergency ophthalmic care particularly in rural communities are a problematic situation that complicates and sets burdens for ophthalmic care delivery. This problem has been investigated widely in the USA, where the EDs are usually inaccessible or without the proper specialists and equipment for ophthalmic emergencies. In these studies, teleophthalmology is considered of high interest and perceived value for remote triage and consultations for both patients and health professionals [92,93].

The COVID-19 pandemic has resulted in altered hospital environments, with decreased outpatient activity and patients unwilling to visit the hospital. This situation forced the development of teleophthalmology so that emergencies are timely referred to and continuously managed. A video consultation platform “Attend Anywhere” in Moorfields Eye Hospital's accident and ED (London, UK) had thriving results for patient satisfaction, consultation duration and waiting time [94]. Additionally, in India, Murthy et al. demonstrated the use of teleophthalmology for acute ocular pain in the times of the COVID-19 pandemic, in order to decrease unnecessary hospital visits and provide guidelines to the practicing ophthalmologists for teleconsultation [95]. Teleophthalmology is now recognized as a tool not only for the pandemic era but also for facilitating ophthalmic care in general.

 6. *Neuro-ophthalmology*

For neuro-ophthalmology, a subspecialty that relies on clinical examinations, the COVID-19 era has been challenging. In a survey conducted by Moss et al. [96], out of 208 practicing neuro-ophthalmologists, the majority of whom increased the use of teleophthalmology due to COVID-19 restriction, characterized access, continuity and patient efficiency of care as benefits and data quality and inaccuracy of fundoscopy as barriers of teleophthalmology. Video telehealth was considered most helpful for cranial nerve palsies, migraine with aura and optic neuritis and not helpful for various optic neuropathies. Grossman et al. [97] referred to the future implications of teleophthalmology in neuro-ophthalmology, highlighting the use in education for practicing residents for video-based teaching, evaluation and distance barrier solving.

Video visits, phone visits, online portal communication, provider-to-provider Consultation (“E-consult”), home testing with a smartphone for color vision and tablet for perimetry are all implementations of teleophthalmology that have been investigated and are utilized in neuro-ophthalmology practices, without replacing but facilitating clinical practice [98].

Challenges to overcome/limitations

As with every novel technological innovation, teleophthalmology and its implementations do not come without limitations. The benefit of teleophthalmology can be enormous and the possibilities numerous, but barriers exist and need to be addressed by the scientific community. Unfamiliarity with teleophthalmology for both patients and health professionals, as presented in Liu et al., highlighted the need for extensive training and uniform referral standards [45]. A consensus on image gradeability is essential for accurate grading and agreement between the health professionals. Misconceptions about diabetic eye screening and logistical challenges for patients and difficulty in the referral process and the use of teleophthalmology for primary care providers were barriers identified through interviews in a qualitative study in a rural US primary care clinic [11].

Even though minimum technical knowledge and resources for sharing images are needed for virtual consultations, photographs and even video calls do not provide as much information as can be gained by slit-lamp examination and important tests, such as intraocular pressure measurement, are not possible. Furthermore, several other issues, such as informed consent of the patient, payment for the consultation, medico-legal liability for the treating doctor and prescription of medication, need further deliberation and policy formation.

On patients’ perspectives on teleophthalmology survey including willingness to pay (WTP), the cost was cited as a concern for which education is required, particularly in underserved and low-resourced populations [99].

Teleophthalmology can also put additional pressure on healthcare personnel creating the phenomenon or workarounds that maximize the number of tasks for ophthalmologists and increase patient volume. This way, the implementation of teleophthalmology in everyday clinical practice has to be very careful and needs to be adequately adapted in order to achieve the best results (Table 1) [100]. Non-medical healthcare professionals (e.g. trained optometrists, specialist nurses or specialist ophthalmic photographers) could assist with the grading of virtually obtained images and decrease the burden on the limited pool of medically trained staff.

**Table 1:** Various applications of teleophthalmology in different clinical fields and achieved outcomes.

Clinical Field	Teleophthalmology application	Results
Posterior segment pathology	Portable ophthalmic camera system powered by an iOS handheld mobile device [2]	Timely referral
Anterior segment pathology	EyeSmart study-I (Teleconsultation) [86]	Management of anterior segment conditions, bridging the distance gap for rural areas, cataract timely diagnosis
	Smartphone-assisted anterior segment photography with macrolens 10 diopters lens [87]	
Diabetic retinopathy	Smartphone-based retinal photography and portable screening devices [4] (review)	Regular monitoring and population screening
Age-related macular degeneration (AMD)	Mobile app for hyperacuity self-monitoring [56]	Self-testing of AMD
Retinopathy of prematurity (ROP)	Smartphone-guided wide-field imaging [60]	Screening for ROP
	Smartphone-based fundus photography “SROP” [61]	
Retinal pathologies (general)	Portable fundus cameras and smartphone-based fundus imaging systems [9]	Addressing preventive blindness (screening, monitoring)
High intraocular pressure (IOP)/ Glaucoma	“Icare HOME tonometry” [75]	Self-monitoring of variability of IOP
	Smartphone-based head mounted perimeter for detection of visual field defects “GearVision” [76]	Facilitation the detection of visual field defects early
Pediatric eye pathologies	“GoCheck Kids” mobile application [80]	Evaluation of visual acuity
	Video recordings [81]	Diagnosis of pediatric amblyopia and eye disease
Acute ocular pain	Teleconsultation [92]	Early referral, decompression of ophthalmic clinics
Ophthalmoscopy	High-resolution direct ophthalmoscopy with an unmodified iPhone X [77]	Access to basic ophthalmic care without distance barriers
Acute hypertension	Ocular fundus smartphone device (D-Eye, Si14 S.p.A., Padova, Italy) [91]	Assessment of the presence of grade III and IV Keith Wegener retinopathy/ fundoscopic examination

Artifacts can also appear when using fundus photography means for ophthalmology, and even if they are not much documented, they need to be addressed, possibly with optimization of imaging devices so that resulting in false-positive referral is avoided [101].

Future perspectives

The futures perspectives of teleophthalmology are broad and complex. The role of telemedicine in healthcare is expanding and the COVID-19 pandemic has accelerated the implementation of telemedical tools, especially in ophthalmology. Ophthalmologists are already embracing the novel technology while observing its benefits through the pandemic. Successful devices’ use and programs implementation require carefulness and detailed research to overcome barriers concerning the cost-effectiveness and the ethical concerns for the acquired data [5,102].

Demographic and market requirements will also play an important role in telemedicine applications acting as guidance markers for additional research. Innovation, robust programs and improvement of existing devices are supposed to positively impact VA outcomes in visual-threatening diseases like DR and ROP. Future teleophthalmology could support more personalized management through continuous data recording [103,104].

This data can be used for AI programs that come along with the development of teleophthalmology (Table 2). The combination of AI and teleophthalmology is likely to be revolutionary for ophthalmology when it is accurately integrated into ophthalmological practice [105,106].

**Table 2:** Applications of artificial intelligence in ophthalmology. AI - artificial intelligence

Use	AI system
Diabetic retinopathy screening	Software based on deep learning techniques [31]
Diagnosis of plus and pre plus disease in an independent test set of 100 retinal images	Algorithm based on deep learning to automatically diagnose plus disease from retinal photographs [65, 66]
Effective triage of the patients and facilitation of glaucoma clinical practice (not yet approved)	AI with deep learning algorithm [78]
Assessment and diagnosis of adult and pediatric cataracts of varying severities	Deep learning algorithm, “ResNet” [89]

## Conclusions

As the COVID-19 pandemic is still ongoing, the need for change and adaption of medical practice to the new circumstances is undoubtable. Teleophthalmology addresses several eye pathologies that otherwise could be overlooked, late diagnosed or incorrectly managed. Teleophthalmological devices and programs are being implemented and researched widely and have been demonstrating positive results. With the contribution of AI to teleophthalmological approaches studies are showing that this could be the future for ophthalmological practice.

Performance validation, safe data acquisition and economic feasibility constitute the main concerns for the implementation of teleophthalmological tools, while the benefits are steadily being recognized. Retinopathies, glaucoma, and age-related macular degeneration are being in the spotlight of teleophthalmological research so that visual impairment is prevented. Eye care delivery systems offering high-quality patient care will have to consider the movement of ophthalmological care from the doctor’s office to the patients’ homes. Toward the goal of providing the best possible ophthalmological care and reducing preventable visual impairment of any form, further studies need to be constructed and funded.
